# Low Cost Simulator for Heart Surgery Training

**DOI:** 10.5935/1678-9741.20160089

**Published:** 2016

**Authors:** Roberto Rocha e Silva, Artur Lourenção, Maxim Goncharov, Fabio B. Jatene

**Affiliations:** 1 Instituto do Coração do Hospital das Clínicas da Faculdade de Medicina da Universidade de São Paulo (InCor-HCFMUSP), São Paulo, SP, Brazil.

**Keywords:** Cardiovascular Surgical Procedures, Education, Training

## Abstract

**Objective:**

Introduce the low-cost and easy to purchase simulator without biological material so that any institution may promote extensive cardiovascular surgery training both in a hospital setting and at home without large budgets.

**Methods:**

A transparent plastic box is placed in a wooden frame, which is held by the edges using elastic bands, with the bottom turned upwards, where an oval opening is made, "simulating" a thoracotomy. For basic exercises in the aorta, the model presented by our service in the 2015 Brazilian Congress of Cardiovascular Surgery: a silicone ice tray, where one can train to make aortic purse-string suture, aortotomy, aortorrhaphy and proximal and distal anastomoses. Simulators for the training of valve replacement and valvoplasty, atrial septal defect repair and aortic diseases were added. These simulators are based on sewage pipes obtained in construction material stores and the silicone trays and ethyl vinyl acetate tissue were obtained in utility stores, all of them at a very low cost.

**Results:**

The models were manufactured using inert materials easily found in regular stores and do not present contamination risk. They may be used in any environment and maybe stored without any difficulties. This training enabled young surgeons to familiarize and train different surgical techniques, including procedures for aortic diseases. In a subjective assessment, these surgeons reported that the training period led to improved surgical techniques in the surgical field.

**Conclusion:**

The model described in this protocol is effective and low-cost when compared to existing simulators, enabling a large array of cardiovascular surgery training.

**Table:** 

**Abbreviations, acronyms & symbols**	
ASD	= Atrial septal defect
EVA	= Ethylene vinyl acetate

## INTRODUCTION

Young medical students and surgeons have been trained in cadavers and live animals since the 19^th^ century. Currently, due to the difficulties to obtain and use cadavers, ethical issues and significant anatomic differences between animals and humans^1^^-^^3^, new methods to teach surgical techniques have been pursued such as the use of artificial simulators^[^^4^^]^.

Simulation may be defined as "the technique that imitates the behavior of a situation or process (be it economic, military, mechanical, etc.) by means of an analogous situation or device, specifically dedicated to study or personal training"^[^^5^^,^^6^^]^; it may also be simply referred to as a "technique that uses a simulator as an object of partial or total representation of a test to be replicated"^[^^7^^]^.

The validity of simulations as a medical learning process has been scientifically investigated^[^^5^^,^^7^^-^^10^^]^. Research involving medical learning and teaching using simulation is difficult to interpret, especially because one cannot be sure whether the method has made any difference or whether those using the method were more dedicated^[^^7^^]^.

Medical students have reached similar results in surgical techniques using simple or sophisticated simulators^[^^11^^,^^12^^]^ and any training using simulators produced superior results than theoretical training alone^[^^5^^]^.

There are different models of simulators as proposed by Andrade^[^^13^^]^, who used a dummy and an anatomic specimen (heart): the model presented by Corso et al.^[^^14^^]^ in the 41^st^ Brazilian Congress of Cardiovascular Surgery 2014 for training in videoassisted surgery, the project produced by Johnson & Johnson, by means of its subsidiary Ethicon, and even models that produce movements, for the training of surgery without cardiopulmonary bypass^[^^15^^]^, among others that are very sophisticated, where anatomic models mimic the consistency and response of live tissues.

In the 42^nd^ Brazilian Congress of Cardiovascular Surgery, we presented a low-cost simulator using a shoebox^[^^16^^]^. The model using a cardboard box caused some problems: a) cardboard could not be used/stored in a hospital environment due to contamination issues and because it obstructed the passage of natural light, requiring a light source; b) it was limited to exercises related to coronary artery disease and simple exercises in the aorta.

We abandoned the original cardboard box, but kept the low-cost concept of the initial project. We propose the use of a transparent plastic box that eliminates the previous contamination and light source problems, and add new methodology and accessories to provide more surgical treatment options, including valve replacement and valvoplasty, atrial septal defect correction and treatment of the aorta.

## METHODS

### Preparation of the Simulator

The material used to manufacture the simulator was purchased in regular stores (stationary stores, office depots, household supply and construction material stores). To better evaluate cost, we separated the materials into permanent materials, which are those required to build the simulator (Table 1) and consumables, which are those used for the different exercises (Table 2). We have excluded surgical suture lines, forceps and needle holders from these amounts, since they have to be used in any training.

**Table 1:** Price of permanent materials used to manufacture the simulator. (Prices obtained in the first semester of 2015).

**Permanent Material**	**Price**
Transparent plastic box with lateral flaps (32 x 22 x 10 cm)	US$ 2.66
Wooden shelf - smooth finishing (40 x 20 x 1.5 cm)	US$ 6.68
Welding cap - 40 mm	US$ 0.45
Welding cap - 25 mm	US$ 0.42
Sewer pipe elbow (50 mm - 45 degrees)	US$ 1.32
Simple sewage pipe sleeve (40 mm)	US$ 0.42
Paper clip - 19 mm (package with 10 units)	US$ 7.07
Elastic bands (package with 110 units)	US$ 1.00
Screw - 4.2 x 13 mm (box with 500 units)	US$ 6.67
Total	US$ 19.20

**Table 2:** Price of materials used in cardiovascular surgery training exercises (obtained in the first semester of 2015).

**Material**	**Price**	**Qty**	**Cost**
Silicone ice tray	US$ 13.90	1	US$ 4.63
Cupcake silicone mold - 7 cm (box with 12 units)	US$ 7.17	12	US$ 7.17
Cupcake silicone mold - 5 cm (box with 12 units)	US$ 7.17	6	US$ 3.59
EVA rubber mats (40 x 60 cm - package with 5 units)	US$ 4.50	1	US$ 0.90
Common rubberized fabric (1.40 m wide - price of the linear meter)	US$ 6.00	10 commission	US$ 0.60
Spaghetti balloon (8-unit package)	US$ 1.92	1 package	US$ 1.92
Total			US$ 18.81

**Fig. 1:**
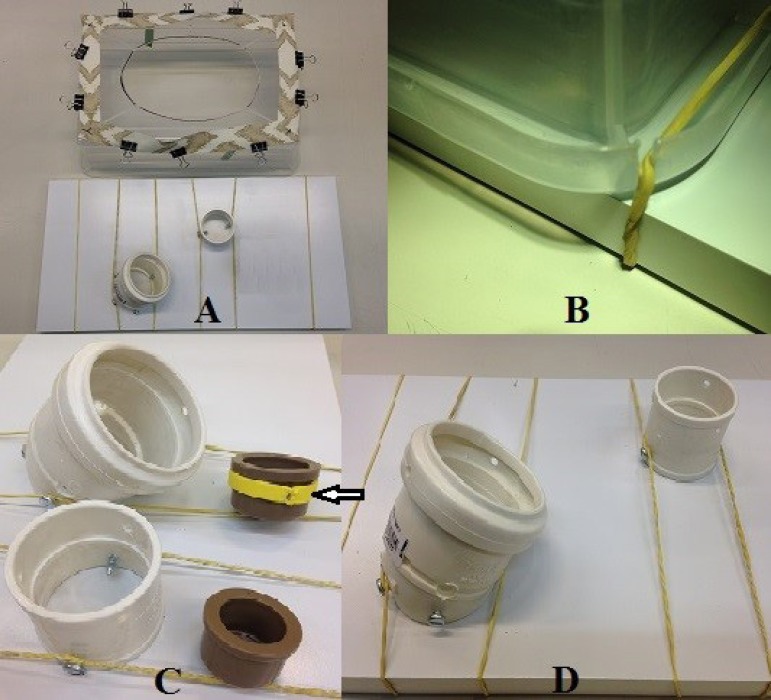
**A.** Transparent plastic box with rubberized fabric straps, smooth shelf with elastic band sand two sewage sleeves, simulating the mitral and aortic rings. **B.** Detailed fixation of the box on the shelf with the support of elastic bands. **C.** Sewer pipe connections simulating mitral and aortic rings with caps representing the prostheses, one of which shows the strap (arrow), which is the place for passing the “prosthesis” stitches. **D.** The same images seen from a different angle.

Manufacture of the simulator was very simple. A 20 cm width, 30 cm length, 10 cm height transparent plastic box was used for each individual simulator. We used a soldering iron to produce an oval 25 x 18 cm opening on the bottom of the box to simulate an anterior thoracotomy (Figure 1A). The box was then placed bottom up on a wooden shelf. Four grooves were created with the soldering iron on the bottom corners of the box. Elastic bands held the box to the shelf by these corner grooves (Figure 1B).

Next to the four angles of the surface around the "thoracotomy", 2 mm holes were made and through them we fixed approximately 2.5 cm wide rubberized fabric straps along the full extension of the surface (Figure 1A). These straps hold the suture wires with 19 mm paper clips during training, whereby Kelly forceps are therefore not required and, in some cases, even assistants are not required.

### Exercises

The structure providing the base for the aortic valve simulation is a simple 40 mm sewer pipe sleeve. At the lower end, two holes are punched on opposite sides where 4.2 x 13 mm bolts are screwed in, which are used to attach this structure to the wooden base with elastic bands. At the upper end, three 4 mm holes are uniformly distributed (Figure 1). These holes are used to stich 5 cm silicone molds using cotton thread. Once they are put in place, these molds simulate the aortic valve.

**Fig. 2:**
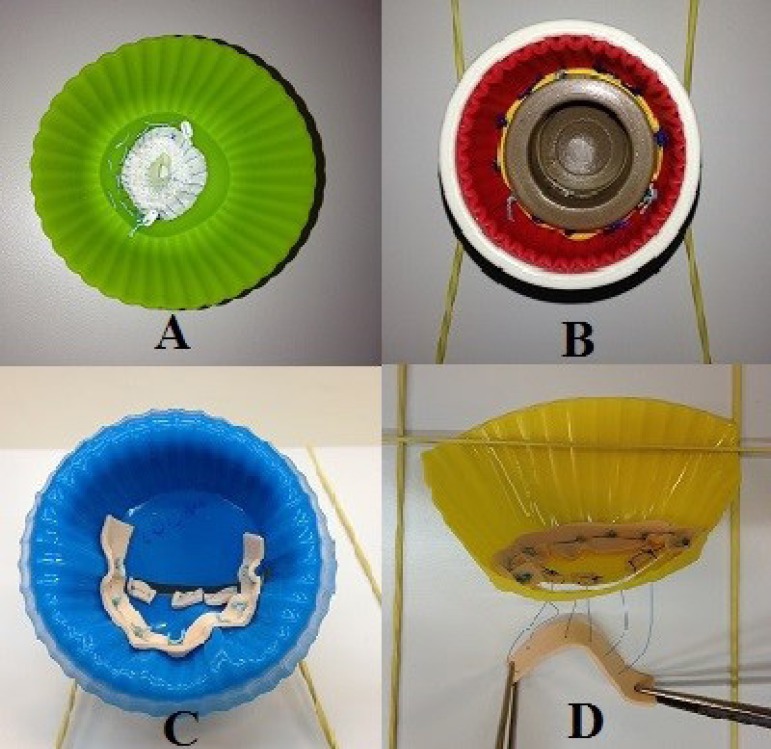
**A.** Upper view of the silicone mold in tricuspid valvuloplasty exercise, ASD and proximal anastomosis. **B.** Upper view of the aortic prosthesis implantation exercise results. **C.** Upper view of the silicone mold in mitral valvuloplasty exercise. **D.** Lateral view of the silicone mold in a simulation practice for making the mitral valve neochordae (half the mold wall was removed to facilitate exposure).

**Fig. 3:**
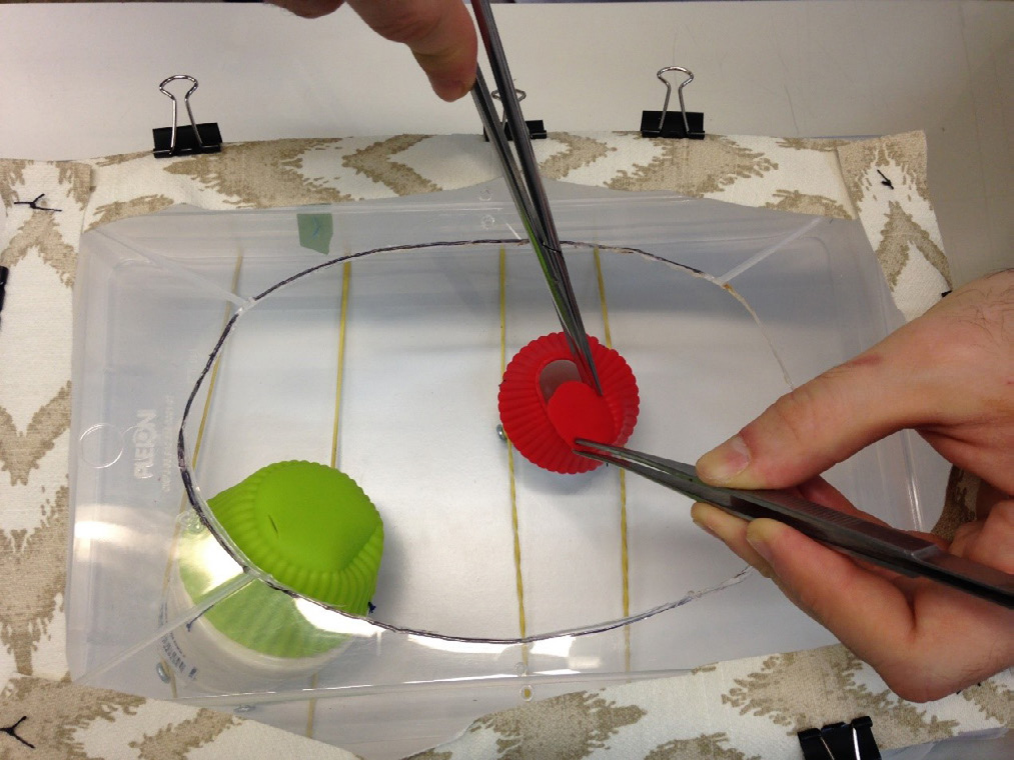
Removing the silicone mold bottom for an ascending aorta replacement exercise.

For the simulation of the mitral valve, a 50 x 45 mm sewer pipe elbow is used. The finer end is cut at the bend so that the other end makes an angle with the wooden surface. On the lower end, which is cut, four opposite 4 mm holes are made for 4.2 x 13 mm bolts, which are used to attach this structure to the wooden base with elastic bands. At the upper end, three 4 mm holes are uniformly distributed (Figure 1). These holes are used to stitch 7 cm silicone molds using cotton thread. Once they are put in place, these molds simulate the mitral valve. Mitral valve repair may also be performed (Figure 2A) with mitral neo-chordae grafts (Figure 2B). For the training of both the valvar prosthesis graft and the aortic aneurysm repair, the bottom of the molds is resected (Figure 3).

For the simulation of aortic and mitral prosthesis, respectively 25 and 40 mm welding caps are used. An ethylene-vinyl acetate (EVA) strap is attached to the base of each cap to simulate the prosthesis rings. Each type of prosthesis simulator is used in its own respective aortic and mitral valve simulator (Figure 1C). Figure 2C shows an implanted aortic prosthesis.

**Fig. 4:**
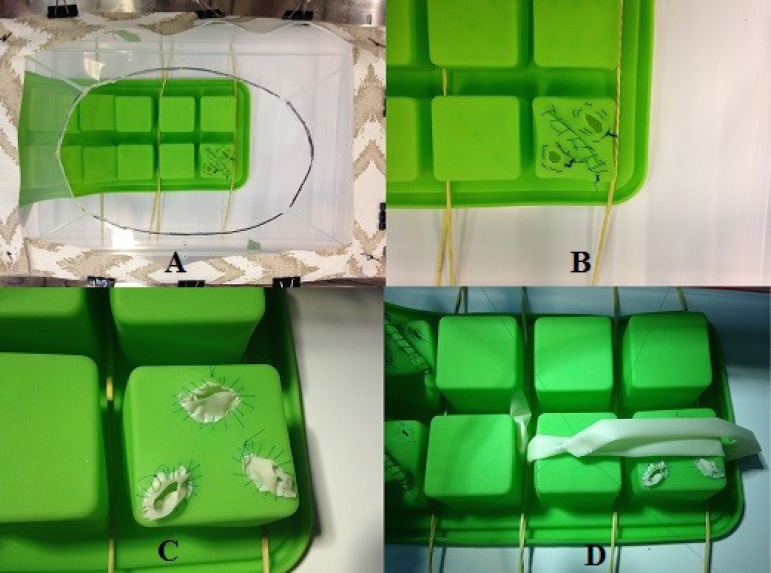
**A.** Ice tray used to train aortotomy, aortorrhaphy, and preparation for aortic cannulation. **B.** The same aspect from a closer look. **C.** Details of coronary proximal suture using spaghetti balloon drain. **D.** The spaghetti balloon simulating a coronary artery bed, for the practice of distal sutures.

For the training of aortotomy, aortorrhaphy, coronary proximal and distal anastomoses, silicone ice trays and spaghetti party balloons are used. The ice trays are attached to the wooden base with the elastic bands. Their surfaces simulate the aortic rim for training purposes (Figures 4A and 4B). The spaghetti balloons simulate the vein for proximal (Figure 4C) and distal (Figure 4D) anastomoses.

**Fig. 5:**
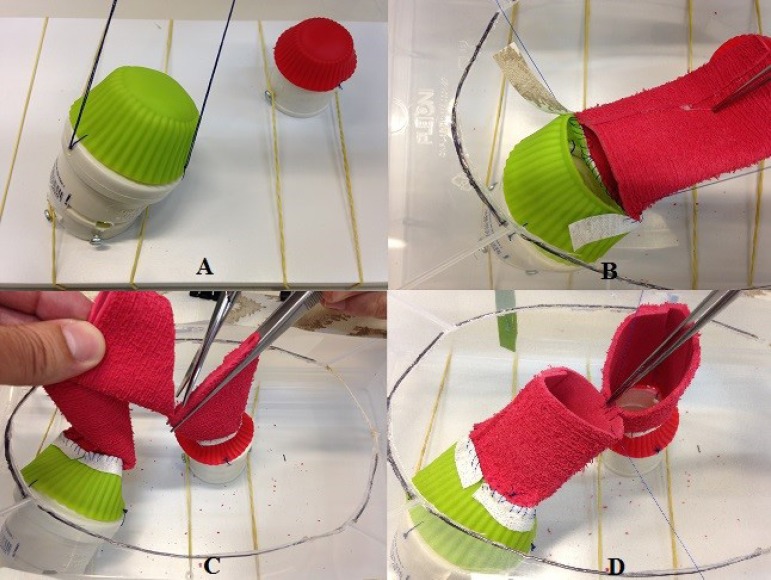
The photos show four sequential steps of the aorta replacement exercise. **A.** Stitching of an inverted silicone mold onto sewer pipe connections. **B.** Suture of EVA pipe with fabric strap support simulating distal anastomosis. **C.** Resecting the excessive EVA pipe. **D.** EVA pipe end-to-end anastomosis.

To simulate the aorta in aortic aneurysm repair training, the mitral and aorta simulator is used; however, the silicone is inverted (Figure 5A). The aortic tube to be grafted is made from EVA fabric. This fabric is cut and rolled to the desired diameter, and then attached with hot glue (overlapping rims by 1cm). It will have to be sutured at the proximal (Figure 5B) and distal openings. Coronary ostia regrafting with spaghetti balloon anastomosis on the lateral portion of the inverted silicone mold may also be trained (Figure 5C). Finally, an end-to-end EVA pipe anastomosis may be performed (Figure 5D).

To train closure of atrial septal defect, the same mitral support is used. After the patch suturing exercise, a tricuspid annuloplasty may be subsequently performed using the same model (around the sutured patch).

The trainee alone, under supervision of a senior surgeon, did most exercises. Some exercises needed the help of an assistant who was the senior surgeon or another trainee. There by, each thoracotomy simulator was individual, but could be used to train surgeon and first assistant surgeon simultaneously.

## RESULTS

At our service, training was carried out by means of bench exercises, totaling nearly 22 hours, with supervised exercises every fortnight. Those exercises were later repeated at home or anywhere else, since every resident received an off-laboratory kit to use. The minimum amount of repetitions was set on the basis of exercise difficulty level. From the minimum amount of required exercises, we then calculated the total consumables used (Table 2).

There was training on aortorrhaphy, aorta purse string suture, proximal and distal coronary anastomoses, valve disease treatment, aortic disease repair, and atrial septal correction. We noticed that the repetition of these exercises facilitated the performance of residents on a surgical field. Residents evaluated this subjectively after the end of their training period.

The exercises were performed in a well-lit setting, without the need for a surgical light source.

The straps at the edge of the box providing support to the paper clips enabled training with reduced surgical materials, such as Kelly forceps, and in some cases avoiding the help of an assistant, thus providing for reduced costs and easy training anywhere, including home. Total cost per resident was US$ 38.01, where permanent materials were estimated at US$ 19.20 (Table 1) and consumables, at US$ 18.81 (Table 2) (prices collected in the first semester of 2015).

## DISCUSSION

In spite of how easy and cheap it was to obtain the simulator, our first one could not be used in a hospital setting because it is subjected to contamination. Since the walls were opaque, there was need for a surgical light source, which made it more difficult to use in any setting.

The solution found for that problem was to adopt a transparent plastic box, which is easy to find in any retail household shop. And it helped eliminate the need for a light source, both in and out of the laboratory.

In 2015 first semester values, the cost to make each exercise kit was below US$ 40, which is affordable to any institution. If compared to simulators found in the international market, this cost was up to 100 times cheaper. Furthermore, we should always consider that some items, like the minimum sellable amounts of straps and bolts cater to the needs of many simulators, which virtually writes off their cost. The use of straps with fixing points eliminated the use of part of the surgical instrumentation, which contributed to further reducing the institution's training project costs.

Along with the new "thorax", we added a number of other exercises, such as valve replacement and valvoplasty, atrial septal defect correction, aorta replacement, and we also enhance "coronary" anastomoses. These exercises were cheerfully welcomed by the young surgeons, who developed their skills in the use of some techniques they only knew from theoretically.

## CONCLUSION

The model described in this protocol is effective and lowcost when compared to existing simulators, enabling a large array of cardiovascular surgery training.

**Table:** 

**Authors' roles & responsibilities**	
RRS	Conception and design study; execution of operations and/ or trials; manuscript writing or critical review of its content; final manuscript approval
ALJ	Execution of operations and/or trials; manuscript writing or critical review of its content; final manuscript approval
MG	Execution of operations and/or trials; manuscript writing or critical review of its content; final manuscript approval
FBJ	Final manuscript approval

## References

[1] Anastakis DJ, Regehr G, Reznick RK, Cusimano M, Murnaghan J, Brown M (1999). Assessment of technical skills transfer from the bench training
model to the human model. Am J Surg.

[2] Carrel A (1910). VIII. On the experimental surgery of the thoracic aorta and the
heart. Ann Surg.

[3] Magalhães HP (1983). Técnica cirúrgica e cirurgia experimental.

[4] Trehan K, Kemp CD, Yang SC (2014). Simulation in cardiothoracic surgical training: where do we
stand?. J Thorac Cardiovasc Surg.

[5] Bradley P (2006). The history of simulation in medical education and possible
future directions. Med Educ.

[6] Ziv A, Wolpe PR, Small SD, Glick S (2003). Simulation-based medical education: an ethical
imperative. Acad Med.

[7] Pazin A, Scarpelini S (2007). Simulação: definição. Medicina (Ribeirão Preto).

[8] Dillon GF, Boulet JR, Hawkins RE, Swanson DB (2004). Simulations in the United States Medical Licensing Examination
(USMLE). Qual Saf Health Care.

[9] Moorthy K, Munz Y, Sarker SK, Darzi A (2003). Objective assessment technical skill in surgery. BMJ.

[10] Vozenilek J, Huff JS, Reznek M, Gordon JA (2004). See one, do one, teach one: advanced technology in medical
education. Acad Emerg Med.

[11] Denadai R, Souto LR (2012). Organic bench model to complement the teaching and learning on
basic surgical skills. Acta Cir Bras.

[12] Anastakis DJ, Regehr G, Reznick RK, Cusimano M, Murnaghan J, Brown M (1999). Assessment of technical skills transfer from the bench training
model to human model. Am J Surg.

[13] Andrade JCS (1994). Nova metodologia para ensino e ensaio de técnicas
operatórias em cirurgia cardíaca. Rev Bras Cir Cardiovasc.

[14] Corso RB, Silva AA, Lima EB, Goulart AH (2014). Simulador para cirurgia cardíaca mini-invasiva
vídeo-assistida. Apresentação de protótipo
desenvolvido e utilizado para treinamento em nosso
Serviço. Anais.

[15] Hammon JW, Crawford FA, Members of the Senior Tour (2014). The characteristics of good thoracic surgical
training. J Thorac Cardiovasc Surg.

[16] Rocha e Silva R Treinamento cirúrgico simples para procedimentos
cardiovasculares simples e complexos. Anais.

